# Neuropsychological Functioning and Temperament Traits in a Czech Sample of Children and Adolescents at Familial Risk of Bipolar Disorder

**DOI:** 10.3389/fpsyt.2019.00198

**Published:** 2019-04-09

**Authors:** Michal Goetz, Tomas Novak, Michaela Viktorinova, Radek Ptacek, Marketa Mohaplova, Antonin Sebela

**Affiliations:** ^1^Second Faculty of Medicine, Charles University Prague, Praha, Czechia; ^2^Department of Child Psychiatry, Motol University Hospital, Praha, Czechia; ^3^National Institute of Mental Health, Klecany, Czechia; ^4^Third Faculty of Medicine, Charles University Prague, Prague, Czechia; ^5^First Faculty of Medicine, Charles University in Prague, Prague, Czechia

**Keywords:** bipolar disorder, offspring, neuropsychological functioning, temperament, at risk

## Abstract

**Background:** Although a positive family history is the strongest predictor for bipolar disorder (BD), most offspring of BD parents (BO) will not develop the disorder. Identification of vulnerability markers for BD is essential for specific individual risk estimation. Impairments in cognitive functioning and the presence of specific temperament traits are considered promising candidates.

**Methods:** Sixty-three BO (48% female; 11.8 ± 3.3 years) and 54 control offspring (CO; 44% female; 12.3 ± 3.2 years) comparable in sex (*p* = 0.4) and age (*p* = 0.4) were enrolled. Detection of current sub/threshold mood symptoms by the Kiddie Schedule for Affective Disorders and Schizophrenia and General Behavior Inventory was applied to separate BO into ultrahigh-risk (UHR) and high-risk (HR) subgroups. Cognitive functions were tested by the Developmental Neuropsychological Assessment II test battery, d2 Test of Attention, and Amsterdam Neuropsychological Tasks. Temperament was assessed by the Temperament in Middle Childhood and Early Adolescent Temperament Questionnaires.

**Results:** The BO sample consisted of 5 BD, 17 UHR, and 41 HR participants. We did not observe any significant differences between the BO and CO groups or between the UHR, HR, and CO subgroups (Hedges' *g* = 0.21–0.39) in cognitive functioning. The BO differed significantly in some temperament traits from the CO (*g* = 0.42–0.61), while the UHR subgroup exhibited lower effortful control and attention focusing than both HR and CO participants (*g* = 0.92–1.19).

**Limitations:** The cross-sectional design and wide age range of the sample limited our findings.

**Conclusions:** Neuropsychological impairment does not seem to be a trait marker of BD in the premorbid stage. Temperament with low effortful control and low attention focusing might be associated with the development of mood disorders in BO.

## Introduction

Children of parents with bipolar disorder (BD), i.e., bipolar offspring (BO), have an increased risk of developing the disorder than offspring of mentally healthy parents (control offspring; CO), with an estimated heritability of 59% (1). The clinical staging model for BD was developed to improve early interventions and to prevent its onset (2, 3). BO with no symptoms of mood disorders can be classified in the high-risk (HR) stage, whereas those with subclinical and clinical unipolar mood symptoms can be classified in the ultrahigh-risk (UHR) stage. Despite the fact that a positive family history is the strongest predictor for BD, most BO will not develop the disorder (4). Identifying early markers of vulnerability in both HR and UHR offspring is essential for precise person-level risk estimation (5). Regarding potential markers of risk for BD development in the HR population, both deficits in cognitive functioning and accentuated specific temperament traits have been discussed (6, 7).

A meta-analysis of 42 neuropsychological studies of adult patients with BD in the euthymic phase showed impairment across all neuropsychological domains with effect size values in the moderate-to-large range (Cohen's *d* = 0.5–0.8) (8). Considering the prevalence of neurocognitive deficits in adults with BD, neuropsychological functioning in bipolar youth has also begun to be investigated. A meta-analysis of 24 studies of neuropsychological functioning in children and adolescents with BD in the euthymic phase showed that the BD group, compared with a healthy control group, was significantly impaired in verbal learning, verbal memory, working memory, and visual memory and learning (moderate-to-large effect sizes, Hedge's *g* = 0.76–0.99). However, no statistically significant difference was observed in the domains of attention, vigilance, problem reasoning and solving, and processing speed (9).

There is still a dearth of literature, high heterogeneity in the results and unanswered questions regarding neuropsychological functioning in the offspring of parents with BD. Bora and Özerdem (10) conducted a meta-analysis of 18 neuropsychological studies of offspring or siblings of patients with BD aged 10–25 years. The authors reported aggregated significant, but modest, differences in sustained attention (*d* = 0.36), visual memory (*d* = 0.35), intelligence quotient (*d* = 0.29), processing speed (*d* = 0.26), social cognition (*d* = 0.23), and verbal memory (*d* = 0.21). Furthermore, the lack of relevant information on the presence of subthreshold mood symptoms among participants and their effects on cognitive performance and the frequent use of non-comprehensive neuropsychological testing batteries were mentioned as considerable limitations to current knowledge in this field.

Temperament is defined as a relatively stable predisposition of particular behavior tendencies (11). Specific temperament traits, such as cyclothymic, hyperthymic, and irritable-explosive have been repeatedly described in adults with well-defined BD (12, 13). Tillman et al. (14) reported higher novelty seeking in a sample of children and adolescents with BD than in controls. Temperament characteristics were also studied in a population of BO. Chang et al. (15) found a lower activity level, a higher trend to approach new situations, and a higher trend to follow the same daily sleeping patterns in the BO cohort than in the US normative sample. Higher emotionality in BO than in CO was found in the study of Duffy (16). Singh et al. (17) reported that BO had a lower activity level and a higher trend to regular daily habits than the US normative sample. Recently, Kim et al. (18) showed that BO had a higher level of inhibited temperament than the controls.

In the present study, we used a broad battery of neuropsychological tests in combination with well-established temperament questionnaires to compare cognitive functioning and temperament traits in BO and CO. Furthermore, we applied the clinical staging model of BD and separated the BO group into HR and UHR subgroups to assess the effect of subthreshold mood symptoms (19). Based on the initial literature research, our hypotheses were as follows: (1) BO will have deficits in verbal memory, processing speed, and sustained attention, as well as disruptions in social cognition, in comparison with CO; (2) BO will have higher extraversion and lower effortful control than CO; and (3) there will be a continuum of test performance from UHR to HR to CO, in which CO will perform best.

## Materials and Methods

The study was approved by the ethics committee of Second Faculty of Medicine, Charles University in Prague and the ethics committee of National Institute of Mental Health, Klecany. Written, informed consent from parents and offspring regarding the study protocol were obtained. All procedures performed in this study were in accordance with the ethical standards of the 1964 Helsinki Declaration and its later amendments or comparable ethical standards.

### Recruitment, Inclusion and Exclusion Criteria

Parents with children aged 6–18 years from the clinical register of adults with BD at the National Institute of Mental Health in Klecany, Czech Republic were invited to participate in the study. This register includes adults with BD Type I or II. Patients from the BD register participated in various genetic and neuroimaging studies (20, 21). The control group (CO) was recruited via advertisements placed in local primary and secondary schools. Exclusion criteria for the parents of the CO group were history of any psychiatric disorder and any prescribed psychotropic medications. For both the BO and CO groups, the exclusion criteria were intellectual disability (IQ under 70), sensorimotor disorders, major physical disabilities, and a history of complicated prenatal or perinatal development.

#### BO Subgroups

(a) Offspring who had been diagnosed with bipolar spectrum disorder (BD I, BD II, BD NOS, cyclothymia). (b) The UHR offspring who did not have manic or hypomanic symptoms, and had subthreshold or threshold depressive symptoms. (c) The HR offspring who did not have manic or hypomanic symptoms BD, and did not have subthreshold or threshold depressive symptoms.

### Psychiatric Assessment in Parents and Offspring

#### Psychiatric Interviews

The diagnosis in parents was confirmed with the Schedule for Affective Disorders and Schizophrenia-lifetime version (SADS-L) (22) that was conducted by a board-certified psychiatrist. Parents from the control group were interviewed in the same manner to exclude those with any mental disorder or who were prescribed psychotropic medication. BO, CO, and their parents were interviewed by a board-certified child and adolescent psychiatrist with the Kiddie Schedule for Affective Disorders and Schizophrenia Present and Lifetime Version (K-SADS-PL) (23) to obtain the current psychopathology profile.

#### Assessment of Subthreshold Mood Symptoms

The Czech translation of the General Behavior Inventory-Parent version (GBI-P) was used to screen for mood symptoms (24). The GBI-P contained 73 questions reflecting both the intensity and duration of symptoms and consisted of three subscales: a depression scale, a hypomania/biphasic scale, and a 10-item mania scale (25). Each item on the GBI was rated on a four-point scale of intensity, 0 = “hardly ever,” 1 = “sometimes,” 2 = “often,” and 3 = “very often.” A cutoff score of 16 on the depression subscale was used to determine the presence of subthreshold mood symptoms. This score has a sensitivity of 0.90, a specificity of 0.66, a positive predictive value of 0.75, and a negative predictive value of 0.86 for depressive symptoms in children and adolescents (26).

### Neuropsychological Assessment in Offspring

#### Intellectual Abilities

*Raven's Standard Progressive Matrices* (10–18 years) *and Raven's Colored Progressive Matrices* (6–10 years) were used to estimate general intellectual abilities. This non-verbal test measures abstract reasoning and represents a well-validated tool for assessing the level of fluid intelligence (27). Scaled scores were calculated.

#### Verbal Memory

*List memory* from the Developmental Neuropsychological Assessment battery (NEPSY®-II) (28) is a task in which a participant is required to remember a list of 15 words that are presented over five trials followed by an interference list. Immediate recall after each trial, delayed recall after 20 min and overall memory capacity were assessed. A composite score (sum of trials I-V and delayed recall) was calculated for each participant.

#### Verbal Fluency

*Word generation* from the NEPSY®-II assesses verbal productivity. In the first task, participants are asked to think of as many different words as possible during 60 s that begin with a given letter (phonemic fluency). The second task involves generating words from a specific category (semantic fluency). A total number of correctly generated words for each condition was calculated.

#### Psychomotor Speed

*Baseline speed* from the Amsterdam Neuropsychological Tasks (29) is a computer-based test that measures reactions times in response to stimulus change by pressing a button. Z-scores for reaction times and number of omission, commission and false alarm errors were computed. In total, nine different variables were recorded.

#### Attention and Executive Functions

*The d2 Test of Attention* is a widely used measure of both selective and sustained attention (30). It is a paper and pencil task where the participant is asked to cross out as quickly as possible all target letters (“d”) that are interspersed with non-target letters in a given time. Sustained attention is defined as a sum of correctly identified targets converted to scaled scores. In addition, the percentage of errors and the total number of processed items (overall performance) expressed by scaled scores were also computed. In the present study, this test was not administered to 1 child with dyslexia and to children younger than 8 years who could have problems with letter discrimination.

*Feature identification* from the Amsterdam Neuropsychological Tasks (ANT) is a computer-based test of visuospatial skills, sustained attention and executive functions (inhibition, working memory). Participants are presented with matrix patterns and are asked to decide whether the stimuli are similar to the target pattern. Z-scores of reaction times and omission, commission and false alarm errors were assessed. In total, twenty-five different variables were recorded.

*Shifting attentional set* from the ANT is another computer-based test focusing on sustained attention and the ability to shift and inhibit attention. It contains three different tasks where the first one requires compatible responses, the second one requires incompatible responses and the third one switches between those two types of responses. Z-scores of reaction times and omission, commission and false alarm errors were assessed in each task. In total, thirty-three different variables were recorded.

The *animal sorting* task from the NEPSY®-II is a card-sorting task that measures concept formation. Participants are asked to sort a group of eight cards into two categories based on a rule. Low scores are interpreted as signs of poor initiation, impaired self-monitoring and limited cognitive flexibility. Scaled scores were calculated for each participant.

#### Social Cognition

The *affect recognition* task from the NEPSY®-II was used to assess social cognitive skills and encompassed four separate parts. In the first part, participants were asked to decide whether two photographs of children's faces showed the same emotion (happy, sad, neutral, fearful, angry, and disgusted faces). The second part consisted of choosing a pair of faces that displayed the same affect from a choice of four faces. In the third part, participants selected one face that matched the affect on a presented stimulus face. In the fourth part, participants were shown an emotional face for 5 s and then asked to select two faces from a choice of five that depicted the same emotion. Raw scores from all parts were added up to obtain a scaled total score. Low scores indicated impairments in recognition of facial affect, which has further implications in socioemotional functioning.

#### Temperament

The *Temperament in Middle Childhood Questionnaire (TMCQ*) was used for the assessment of temperament traits in children aged 7–10 years (31). The respondent in this case was the caregiving parent. The parent was asked to rate his or her children using a 5-point Likert scale (ranging from “almost always untrue” to “almost always true”) on 157 questions. The final score for each temperament trait was computed as the sum of scores of trait-specific questions divided by their quantity. For the purpose of our study, the TMCQ was also used in children aged 6–7 years old. *The Early Adolescent Temperament Questionnaire-Revised (EATQ-R)* was used to measure dimensions of temperament in children older than 10 years (32). This instrument consists of 103 self-report items. Individuals were asked to rate themselves using a similar Likert scale as in the TMCQ. Ten dimensions of temperament, which are included in both questionnaires, were analyzed; thus, parent reports and offspring self-report assessments were combined. A combined analysis of parent-reported and offspring self-reported data had been performed in a study by Duffy and colleagues (16). The analyzed temperament traits were as follows: activity level (high scorers likely participate in activities requiring high levels of physical activities), high intensity pleasure/surgency (high scorers have great pleasure or enjoyment related to situations involving high stimulus intensity or novelty), low intensity pleasure (high scorers have great pleasure or enjoyment related to situations involving low stimulus intensity, rate, complexity, novelty, and incongruity), shyness (higher scorers have high behavioral inhibition to novelty and challenge, especially social), attention focusing (high scorers have high capacity to maintain attention as well as to shift attention when desired), inhibitory control (high scorers have high capacity to plan and to suppress inappropriate responses under instructions or in novel or uncertain situations), activation control (high scorers have high capacity to perform an action when there is a strong tendency to avoid it), perceptual sensitivity (high scorers have high detection of slight, low-intensity stimulation in the environment), fear (high scorers exhibit a higher amount of unpleasant affect related to the anticipation of distress), frustration (higher scorers exhibit a higher amount of negative affect related to the interruption of ongoing tasks or goal blocking), and affiliation (high scorers have a high desire for warmth and closeness with others, independent of shyness or extraversion).

The dimensions of activation control, attention focusing, and inhibitory control constitute the higher-order temperament factor of effortful control, whereas high intensity pleasure/surgency, activity level and shyness form the high-order factor of extraversion (33).

#### Procedure

The assessment took place in a quiet room for 1 day and was performed by a boarded child clinical psychologist. The order of tests was the same for all participants: the d2 Test of Attention (9–17 years), list memory (I-VI trials), baseline speed, feature integration, shifting attentional set, list memory delayed recall, word generation, affect recognition, animal sorting and Raven's progressive matrices. Temperament questionnaires were administered after the neuropsychological examination.

### Statistical Analyses

#### BO vs. CO

The demographic and clinical characteristics were compared using unpaired *t*-tests or Fisher's exact tests as appropriate. Neuropsychological data were analyzed by unpaired *t*-tests or Mann-Whitney tests based on the result of the Shapiro-Wilk test for normality. Temperament data were analyzed in the same manner as neuropsychology data.

#### UHR vs. HR vs. CO

The demographic and clinical characteristics were compared using the Pearson chi-square test, one-way ANOVA, Welch's ANOVA, or Kruskal-Wallis test as appropriate. *Post-hoc* Tukey's tests, Games-Howell's tests, or Mann-Whitney tests were administered for variables with observed statistically significant between-group differences. Neuropsychological and temperament data were analyzed in the same manner. The standardized effect size of each intergroup difference (BO vs. CO, and UHR vs. HR vs. CO) was calculated as Hedge's g. Bonferroni's correction for multiple testing was applied to avoid false positive results (0.05/9 for neuropsychological tests except the ANT tests, 0.05/68 for the ANT tests, and 0.05/11 for temperament outcomes).

## Results

### Sample Characteristics

In total, 63 BO from 47 families (parents: 28 cases of BD type I; 19 cases of BD type II) were enrolled to participate together with 54 CO from 43 families. The 33 females and 30 males from the BO ranged in age from 6.2 to 17.9 years (mean: 11.8 ± 3.3). The BO and CO did not significantly differ in sex or age.

### Current Psychopathology

Five cases of bipolar spectrum disorder were found in the BO group (2 BD II, 2 BD NOS and 1 cyclothymia), and no cases of BD were found in the CO group. Seven cases of unipolar mood disorder were found in the BO group (5 major mood disorder, 1 depression NOS, and 1 dysthymia), and no cases of unipolar mood disorder were found in the CO group. Regarding the General Behavior Inventory scores, the BO significantly differed from the CO on all subscales. Subthreshold or threshold mood symptoms were observed in 17 BO and 1 CO, and these results formed the groups of 17 UHR offspring, 41 HR offspring and 54 CO. Between-group differences in age and sex did not reach statistical significance. For more information, see Tables 1, 2. Extended results of the lifetime and current psychopathological profiles of the CO and BO, as well as parental characteristics, are detailed elsewhere (34).

**Table 1:** Demographic and current clinical variables of the BO and CO groups.

	**BO (*N* = 63)**	**CO (*N* = 54)**	***p*-Value**
Female/Male	30/33	24/30	0.4^a^
Age, mean (SD)	11.8 (3.3)	12.3 (3.2)	0.4^b^
PSYCHOPATHOLOGY^*^, ***N*** **(%)**			
Bipolar spectrum disorder^1^	5 (7.9)	0 (0.0)	0.04^a^
Unipolar mood disorder^2^	7 (11.0)	0 (0.0)	0.01^a^
Anxiety disorder^3^	26 (41.3)	4 (7.4)	< 0.001^a^
Substance use disorder^4^	5 (7.9)	0 (0.0)	0.04^a^
ADHD	14 (22.2)	2 (3.7)	0.003^a^
Learning disabilities	5 (7.9)	2 (3.7)	0.3^a^
Behavioral disorders^5^	11 (17.5)	1 (1.9)	0.005^a^
No current psychopathology	22 (34.9)	45 (83.3)	< 0.001^a^
**GENERAL BEHAVIOR INVENTORY, MEAN (SD)**			
Depression scale	13.4 (2.1)	1.9 (0.4)	< 0.001^c^
Hypomanic/biphasic scale	7.7 (1.3)	1.4 (0.3)	< 0.001^c^
10-Item mania scale	2.5 (0.5)	0.2 (0.1)	< 0.001^c^
Psychotropic drug naive, *N* (%)	54 (85.7)	54 (100.0)	0.003^a^
Without psychiatric care, *N* (%)	51 (81.0)	54 (100.0)	< .001^a^

ADHD, attention-deficit hyperactivity disorder; BO, bipolar offspring; CO, control offspring; N, number; SD, standard deviation;

a Fisher's exact test;

b Unpaired t-test;

c Mann-Whitney test;

1 Including BD II, BD NOS, and cyclothymia;

2 Including major mood disorder, depression NOS, and dysthymia;

3 Including generalized anxiety disorder, social anxiety, separation anxiety, specific phobia; panic attack and school phobia;

4 Including alcohol, cannabinoids and amphetamines;

5 Including oppositional defiant disorder and conduct disorder;

* *Some individuals meet criteria for more than one diagnosis*.

**Table 2:** Demographic and clinical variables of the UHR and HR subgroups and the CO group.

	**UHR (*N* = 17)**	**HR (*N* = 41)**	**CO (*N* = 54)**	***p*-Value**
Female/Male	9/8	17/24	24/30	0.7^a^
Age, mean (SD)	12.5 (3.9)	11.1 (2.8)	12.3 (3.2)	0.1^b^
**GENERAL BEHAVIOR INVENTORY, MEAN (SD)**				
Depression scale	28.2 (3.2)^*^	3.5 (0.6)^**^	1.9 (0.4)	< 0.001^c^
Hypomanic/biphasic scale	12.5 (2.7)^*^	3.4 (0.6)^*^	1.4 (0.3)	< 0.001^c^
10-Item mania scale	4.6 (1.2)^*^	0.8 (0.2)^**^	0.2 (0.1)	< 0.001^c^

CO, control offspring; HR, high-risk offspring; N, number; SD, standard deviation; UHR, ultrahigh-risk offspring;

a Pearson's chi-square;

b Welch's ANOVA;

c Kruskal-Wallis test;

* Post-hoc test vs. HR and CO, p < 0.001;

** *Post-hoc test vs. CO, p < 0.05*.

### Neuropsychological Assessment

Sixty BO and 42 CO completed full neuropsychological assessment (two participants in the UHR group and one participant in the HR group refused testing). No significant differences were observed in intellectual abilities [115.2 ± 14.4 vs. 116.5 ± 14.2; *t*_(100)_ = 0.20, *p* = 0.65], list memory, both word generation semantic and phonemic, the d2 test, the animal sorting task, and the affect recognition task. Furthermore, no statistically significant difference was observed in the baseline speed task, feature identification task, or shift attentional set task from the ANT.

The highest, but non-significant, standardized effect sizes were found in the *Z*-scores for the number of commission errors in the third part of the shifting attentional test task (Hedge's *g* = 0.39) and the animal sorting task (*g* = 0.28) in which the BO performed better than the CO. Other neuropsychological tests ranged in effect size from 0.06 to 0.21. Detailed information is reported in Table 3. In Table 3, we do not report all variables recorded in the ANT tests; we present those whose between-group differences were anticipated to be the highest.

**Table 3:** Neuropsychological characteristics of the BO and CO groups.

	**BO (*N* = 60)**	**CO (*N* = 42)**	***p***	**Hedge's g**	**Who was better**
Female/Male	28/32	18/24	0.4^a^	NA	NA
Age, mean (SD)	11.8 (3.3)	12.3 (3.1)	0.4^b^	NA	NA
**INTELLECTUAL ABILITIES, MEAN (SD)**					
Raven's progressive matrices^A^	115.2 (14.4)	116.5 (14.2)	0.7^b^	0.09	CO
**VERBAL MEMORY**					
List memory^A^	11.2 (3.0)	10.9 (2.9)	0.7^b^	0.10	BO
**VERBAL FLUENCY**					
Word generation semantic^A^	13.9 (3.3)	14.6 (3.4)	0.2^b^	0.21	CO
Word generation phonemic^B^	16.1 (7.3)	16.8 (7.8)	0.6^b^	0.09	CO
**PSYCHOMOTOR SPEED, MEAN (SD)**					
Baseline Speed^C^					
Reaction time	−0.4 (0.9)	−0.2 (1.3)	0.5^b^	0.18	CO
Number of false alarm errors	0.1 (1.1)	−0.1 (0.8)	0.5^b^	0.20	BO
**ATTENTION AND EXECUTIVE FUNCTIONS**					
d2 test of attention, mean (SD), range^D^					
Overall performance	58.4 (31.6), 1–99	56.7 (28.2), 10–99	0.8^b^	0.06	BO
Errors	69.1 (25.5), 11–100	72.7 (28.3), 7–99	0.6^b^	0.13	BO
Sustained attention	64.2 (30.7), 1–100	65.9 (28.1), 7–99	0.8^b^	0.06	CO
Feature identification^C^					
Reaction time	−0.3 (1.0)	−0.1 (1.0)	0.2^b^	0.20	CO
Number of false alarm errors	0.3 (3.0)	0.0 (2.0)	0.5^b^	0.11	CO
Shifting attentional set, third task^C^					
Reaction time	0.3 (1.4)	0.1 (1.2)	0.6^b^	0.15	BO
Commission errors	0.5 (1.8)	−0.1 (1.0)	0.1^b^	0.39	BO
Omission errors	0.4 (1.5)	0.3 (1.3)	0.6^b^	0.07	BO
Animal sorting^*A*^	9.4 (3.1)	8.6 (2.5)	0.2^b^	0.28	BO
**SOCIAL COGNITION**					
Affect recognition^*A*^	10.5 (3.1)	11.0 (2.0)	0.3^b^	0.18	CO

BO, bipolar offspring; CO, control offspring; NA, not applicable; SD, standard deviation;

a Fisher's exact test;

b Unpaired t-test;

A Scaled score;

B Raw score;

C Z-score;

D *Percentile*.

#### UHR vs. HR vs. CO

No statistically significant differences were found in the analysis of cognitive performance of the UHR, HR and CO groups (Table 4).

**Table 4:** Neuropsychological characteristics of the UHR, HR, and CO groups.

	**UHR (*n* = 15)**	**HR (*n* = 40)**	**CO (*n* = 42)**	***p***
**INTELLECTUAL ABILITIES, MEAN (SD)**				
Raven's progressive matrices^A^	113.1 (13.3)	116.1 (14.9)	116.5 (14.2)	0.7^a^
**VERBAL MEMORY**				
List memory^A^	12.0 (2.6)	10.9 (2.9)	10.9 (2.9)	0.6^a^
**VERBAL FLUENCY**				
Word generation semantic^A^	14.3 (3.8)	13.5 (3.3)	14.6 (3.4)	0.3^a^
Word generation phonemic^B^	18.3 (7.7)	14.4 (6.7)	16.8 (7.8)	0.1^a^
**PSYCHOMOTOR SPEED, MEAN (SD)**				
Baseline speed^C^
Reaction time	−0.7 (0.7)	−0.3 (1.1)	−0.2 (1.3)	0.8^a^
Number of false alarm errors	0.3 (1.1)	0.0 (1.6)	−0.1 (0.8)	0.4^b^
**ATTENTION AND EXECUTIVE FUNCTIONS**				
d2 test of attention, mean (SD), range^D^
Overall performance	68.0 (34.1), 2–99	61.1 (30.1), 1–99	56.7 (28.2), 10–99	0.5^a^
Errors	67.7 (24.4), 18–97	75.1 (22.7), 21–100	72.7 (28.3), 7–99	0.7^a^
Sustained attention	65.5 (32.8), 3–100	65.8 (29.5), 1–99	65.9 (28.1), 7–99	0.6^a^
Feature identification^C^
Reaction time	−0.6 (1.2)	−0.4 (0.8)	−0.1 (1.0)	0.2^a^
Number of false alarm errors	0.0 (0.8)	0.5 (3.1)	0.0 (2.0)	0.6^a^
Shifting attentional set, third task^C^
Reaction time	−0.3 (1.2)	0.3 (1.3)	0.1 (1.2)	0.4^a^
Commission errors	0.2 (0.9)	0.2 (2.0)	−0.1 (1.0)	0.6^a^
Omission errors	0.0 (0.8)	0.4 (1.7)	0.3 (1.3)	0.7^a^
Animal sorting^A^	8.5 (3.3)	10.0 (2.8)	8.6 (2.5)	0.05^a^
**SOCIAL COGNITION**				
Affect recognition^A^	10.8 (1.4)	10.1 (3.0)	11.0 (2.0)	0.04^a^

CO, control offspring; HR, high-risk offspring; SD, standard deviation; UHR, ultrahigh-risk offspring;

a One-way ANOVA;

b One-way Welch's ANOVA;

A Scaled score;

B Raw score;

C Z-score;

D *Percentile*.

### Temperament Traits

A total of 58 BO and 50 CO completed the temperament questionnaires (one participant from the UHR subgroup and four from the HR subgroup refused to complete the questionnaire). Statistical differences between BO and CO were observed in five temperament dimensions. BO had lower scores on the high intensity pleasure/surgency scale (*t* = −3.17; *p* = 0.002); higher scores on the shyness scale (*t* = 3.01; *p* = 0.003), lower scores on the activation control scale (*Z* = −2.59; *p* = 0.009), higher scores on the frustration scale (*t* = 2.63; *p* = 0.01), and lower scores on the activity level scale (*t* = −2.51; *p* = 0.01) than CO. Differences in the high intensity pleasure/surgency and shyness remained significant even after Bonferroni's correction for multiple comparisons. No statistically significant between-group differences were found for the high-order temperament factors extraversion (*t* = −0.780; *p* = 0.4) and effortful control (*Z* = −1.60; *p* = 0.1). Means and standard deviations of temperament trait subscales are reported in Table 5.

**Table 5:** Temperament traits of offspring.

	**BO (*n* = 58)**	**CO (*n* = 50)**	***p*-Value**	**Hedge's *g***
Female/Male	28/30	23/27	0.5^a^	NA
Age, mean (SD)	11.9 (3.2)	12.1 (3.2)	0.7^b^	NA
**TEMPERAMENT TRAIT, MEAN, SD**				
High intensity pleasure/surgency	2.9 (0.6)	3.2 (0.5)	0.002^b^	0.54
Shyness	3.0 (0.7)	2.6 (0.7)	0.003^b^	0.57
Activation control	3.0 (0.7)	3.3 (0.5)	0.009^c*^	0.49
Frustration	3.3 (0.7)	2.9 (0.6)	0.01^b*^	0.61
Activity level	3.2 (0.8)	3.5 (0.6)	0.01^b*^	0.42
Inhibitory control	3.0 (0.5)	3.1 (0.4)	0.3^b^	0.22
Attention focusing	3.3 (0.9)	3.5 (0.6)	0.3^b^	0.26
Perceptual sensitivity	3.1 (0.7)	3.3 (0.6)	0.2^b^	0.30
Low intensity pleasure	3.2 (0.7)	3.3 (0.7)	0.2^b^	0.14
Fear	2.6 (0.8)	2.5 (0.9)	0.5^b^	0.12
Affiliation	3.5 (0.6)	3.6 (0.6)	0.06^c^	0.17
Effortful control	9.3 (1.8)	9.8 (1.1)	0.1^c^	0.33
Extraversion	9.1 (1.3)	9.3 (1.1)	0.4^b^	0.17

BO, bipolar offspring; CO, control offspring; NA, not applicable; n, number; SD, standard deviation;

^a^Fisher's exact test; ^b^Unpaired t-test; ^c^Mann-Whitney test; ^*^Non-significant after Bonferroni's adjustment.

#### UHR vs. HR vs. CO

Statistically significant differences were observed in the high intensity pleasure/surgency scale (*F* = 7.78; *df* = 2,100; *p* = 0.001), effortful control (*H* = 12.03; *df* = 2,100; *p* = 0.002); activation control (*H* = 10.39; *df* = 2,100; *p* = 0.006), activity level (*F* = 5.14; *df* = 2,100; *p* = 0.008), attention focusing (*F* = 5.26; *df* = 2,100; *p* = 0.01), shyness (*F* = 4.28; *df* = 2,100; *p* = 0.02), fear (*F* = 3.96; *df* = 2,100; *p* = 0.02), and frustration (*F* = 3.69; *df* = 2,100; *p* = 0.03). Differences in high intensity pleasure/surgency and effortful control scales remained significant even after the Bonferroni's correction for multiple comparisons. Means and standard deviations of temperament subscales across the three groups are reported in Table 6.

**Table 6:** Temperament scores across the UHR and HR offspring subgroups and CO group with statistically significant between-group differences.

	**UHR (*N* = 16)**	**HR (*N* = 37)**	**CO (*N* = 50)**	***p*-Value**	***Post-hoc***	***p*-Value**	**Hedge's *g***
High intensity pleasure/surgency, mean (SD)	2.6 (0.7)	2.9 (0.5)	3.2 (0.5)	0.001^a^	UHR vs. HR	n.s.	
					UHR vs. CO	0.001^d^	1.08
					HR vs. CO	n.s.	
Effortful control	8.4 (1.4)	9.9 (1.6)	9.8 (1.1)	0.002^b^	UHR vs. HR	0.004^e^	0.97
					UHR vs. CO	< 0.001^e^	1.19
					HR vs. CO	n.s	
Activation control	2.7 (0.7)	3.2 (0.6)	3.3 (0.5)	0.006^b*^	UHR vs. HR	0.02^e^	0.79
					UHR vs. CO	0.001^e^	1.08
					HR vs. CO	n.s.	
Activity level	2.9 (0.8)	3.3 (0.6)	3.5 (0.6)	0.008^a*^	UHR vs. HR	n.s.	
					UHR vs. CO	0.006^d^	0.92
					HR vs. CO	n.s.	
Attention focusing	2.8 (0.8)	3.6 (0.9)	3.5 (0.6)	0.01^c*^	UHR vs. HR	0.01^f^	0.92
					UHR vs. CO	0.02^f^	1.07
					HR vs. CO	n.s.	
Shyness	3.0 (0.8)	3.0 (0.7)	2.6 (0.7)	0.02^a*^	UHR vs. HR	n.s.	
					UHR vs. CO	n.s.	
					HR vs. CO	0.02^d^	0.57
Fear	3.0 (0.7)	2.3 (0.7)	2.5 (0.9)	0.02^a*^	UHR vs. HR	0.02^d^	1.00
					UHR vs. CO	n.s.	
					HR vs. CO	n.s.	
Frustration	3.4 (0.6)	3.1 (0.7)	2.9 (0.6)	0.03^a*^	UHR vs. HR	n.s.	
					UHR vs. CO	0.02^d^	0.83
					HR vs. CO	n.s.	

CO, control offspring; HR, high-risk offspring; N, number; n.s., not significant; SD, standard deviation; UHR, ultrahigh-risk offspring; ^a^One-way ANOVA; ^b^Kruskal-Wallis test; ^c^One-way Welch's ANOVA; ^d^Tukey's test; ^e^Mann-Whitney test; ^f^Games-Howell's test; ^*^Non-significant after Bonferroni's adjustment.

#### *Post-hoc* Pairwise Comparisons

The UHR offspring subgroup scored lower than both the HR and CO groups in effortful control, activation control and attention focusing. Detailed results are reported in Table 6.

## Discussion

The current study compared the neuropsychological functioning and temperament in offspring of bipolar parents and control offspring. Furthermore, we applied the clinical staging model of BD and assessed the effect of subthreshold mood symptoms on one's cognitive performance. Based on previous evidence from studies on children, adolescents and adults with BD, we hypothesized that the BO will differ in specific cognitive domains and temperament traits from the CO. Somewhat surprisingly, the BO did not differ in any of the tested cognitive domains, although they exhibit more psychopathology than the CO.

Most previous studies in this field have assessed preselected cognitive domains. Deficits in executive functions (cognitive flexibility, inhibition) and spatial memory have been reported in some BO studies (35, 36). In the domain of attention, previous results have been inconclusive, with some studies finding normal performance (36), while others have observed impaired functioning in tasks involving sustained attention (35, 37). Likewise, in some studies, adolescent BO did not show any impairment in socioemotional functioning, such as affect recognition or theory of mind (38), while emotion labeling deficits were observed by others (39). Similarly, inconsistency was found throughout reports on impairment in intellectual functioning of BO (40).

The discrepancy across neuropsychological findings could be explained by heterogeneity encountered in both the BO and CO groups. There is a substantially higher prevalence and incidence of psychiatric diagnoses in BO than in CO (41, 42). Therefore, information on current mental health status is important, as it itself may influence cognitive performance (43). In our study, we reported the current psychopathological profile, as well as the presence of subthreshold mood symptoms, while other studies have reported only lifetime profiles (35, 44, 45). Although our BO sample exhibited a high level of psychopathology, impairments requiring pharmacological treatment were observed in < 20% of the BO (with depression as the leading cause).

Different inclusion and exclusion criteria for CO across studies, with some studies excluding a priori any children with psychiatric disorders (38), may also lead to an overestimation of familial BD risk impact on cognitive functioning in the offspring. In our study, we chose a more naturalistic setting, and participants with current psychiatric diagnoses were also included. Another factor of heterogeneity in neuropsychological findings is the mean age of the participants. Robust differences among the BO and CO were found in older samples, with a higher rate of psychopathology (46–48). In the our study, there was no difference in neuropsychological functioning between the BO and CO, which is in agreement with other studies assessing similarly aged children (36, 49, 50).

In our study, there were no statistically significant differences among UHR offspring, HR offspring and CO, which can be interpreted as follows: (1) neuropsychological difficulties do not seem to be a trait marker at this premorbid stage of BD, as no difference were found between BO and CO, and (2) neuropsychological difficulties do not seem to be a state marker of BD during the early stage of the disease as no difference were found between the HR and UHR groups. However, in line with the staging model of BD, cognitive dysfunction could appear later on the natural history of the disease. Thus, it seems to us that neuropsychological assessments offer limited usefulness for early risk estimation in offspring in the UHR or the HR groups regarding BD development.

The second objective of our study was to assess whether the BO differed from the CO in temperament traits. Samples differed only moderately. However, only lower interest in activities involving high intensity or novelty and higher levels of behavioral inhibition to novelty and challenge found in the BO remained significant after correction for multiple testing. When we applied the clinical staging model for BD, the difference in temperament traits across the groups became more apparent. The continuum of effortful control levels observed among UHR offspring, HR offspring and CO fully aligned with our hypothesis. Furthermore, this result reached a large effect size and remained statistically significant after correction for multiple testing. Effortful control is linked to the child's optimal development (51) and plays a central role in the self-regulation of emotion and related processes (52). Impairments in the function of brain regions related to emotional regulation have been repeatedly observed in BD samples (53, 54). Furthermore, low effortful control has been associated with higher externalizing symptoms (55), and externalizing symptoms have a high prevalence in BO offspring in general (56, 57). Our finding of low effortful control in the UHR offspring, in comparison to not only the CO but also the HR offspring, is particularly interesting, as it may be seen as an inherited risk factor for the future development of psychiatric disorders.

We also hypothesized that the BO would have higher levels of extraversion than the CO, but no difference was found in our study. In contrast, the BO (both UHR and HR subgroups) exhibited higher inhibition to novelty and social activities than the CO. Higher levels of depressive symptoms in the BO may partly explain that finding (58). On the other hand, Chang et al. (15) found a higher tendency to approach new situations in their BO sample than in the US normative sample. This opposite finding may account for the cross-cultural differences in child temperament (59).

The current study has several limitations that must be taken into consideration when interpreting the results. The cross-sectional design is less able to detect factors that contribute to the risk of BD onset in the BO, as we were unable to fully eliminate the effect of present psychopathology on temperament due to collinearity. The limited sample size led to unevenly distributed subgroups, which may have underpowered the results. The large age range of the participants is a main limitation of the temperament trait assessment, as temperament is not fully established in children compared with adolescents. We analyzed only temperament traits that were included in both the EATQ-R and TMCQ to mitigate this limitation. The TMCQ was used in four children under 7 years old, although this questionnaire has not been validated in this population, which may have led to a misrepresentation of their temperament. The psychiatrist who interviews the offspring knew the parental status (bipolar vs. healthy), which may have led to a bias due to lack of subject blinding. Finally, the willingness of parents to participate in the study might have reflected their concerns about the mental health of their offspring, increasing the risk of a reporting bias. Despite these limitations, the present study also has strengths. A comprehensive neuropsychological battery was used to evaluate cognitive functioning at the same time as the temperament assessment. The current psychopathology profile was assessed in participants at both the threshold and subthreshold levels, and it was used for application of the developmental staging model of BD. Furthermore, the naturalistic setting of CO inclusion and exclusion criteria make the results more realistic as it prevents the formation of a supercontrol group.

## Conclusion

No significant difference was found in neuropsychological functioning between the BO and CO, suggesting that cognitive impairment is not a trait marker of BD. Furthermore, cognitive impairment does not seem to be a state marker of the premorbid stage of BD, as no significant difference was found between the UHR offspring, the HR offspring and the CO. However, in line with the staging model of BD, cognitive dysfunction could appear later in the natural history of the disease.

On the other hand, compared to other temperament traits, low effortful control and low attention focusing in the BO may be associated with a higher risk of BD development. Further longitudinal research, combined with functional brain imaging, is needed to clarify the usefulness of the assessment of temperament traits of effortful control and attention, focusing on the precise evaluation of the risk of BD development in the HR and UHR populations.

## Author Contributions

MG participated in study design development, data collection, and manuscript preparation. TN participated in data analysis and manuscript preparation. MV participated in the manuscript preparation. RP and MM participated in data collection. AS participated in data analysis and finalization of the manuscript.

### Conflict of Interest Statement

The authors declare that the research was conducted in the absence of any commercial or financial relationships that could be construed as a potential conflict of interest.
